# Association between glutamate transporter gene polymorphisms and obsessive-compulsive disorder/trait empathy in a Korean population

**DOI:** 10.1371/journal.pone.0190593

**Published:** 2018-01-05

**Authors:** Hae Won Kim, Jee In Kang, Eun Hee Hwang, Se Joo Kim

**Affiliations:** 1 Department of Medical Education, Yonsei University College of Medicine, Seoul, Republic of Korea; 2 Institute of Behavioral Science in Medicine, Yonsei University College of Medicine, Seoul, Republic of Korea; 3 Department of Psychiatry, Yonsei University College of Medicine, Seoul, Republic of Korea; Case Western Reserve University Jack Joseph and Morton Mandel School of Applied Social Sciences, UNITED STATES

## Abstract

Accumulating evidence suggests that the glutamatergic system plays a major role in the pathophysiology of obsessive compulsive disorder (OCD) and empathic processing. Particularly, genetic influence of glutamate transporter gene (*SLC1A1*) on OCD has been frequently replicated in previous studies, but several studies did not replicate the result. Therefore, we aimed to replicate the associations between the *SLC1A1* and OCD in a Korean population. In addition, we investigated the influence of *SLC1A1* on trait empathy, impairments in which are characteristic of OCD. Six single-nucleotide polymorphisms (SNP) of *SLC1A1* were genotyped in 615 patients with OCD and 508 healthy controls. The interpersonal reactivity index (IRI)—which consists of four subscales (perspective taking, PT; fantasy seeking, FS; empathic concern, EC; personal distress, PD)—was assessed from 277 patients with OCD and 395 controls. There were no significant associations between OCD and SNPs or haplotypes of *SLC1A1*. Patients with OCD exhibited significantly lower PT and higher PD scores than controls. The C-T-G haplotype at rs301430-rs301434-rs3087879 of *SLC1A1* was significantly associated with higher PD scores after adjusted for age, sex, and OCD status. Our results suggest that six common SNPs of *SLC1A1* may not contribute to the development of OCD, but may contribute to certain aspect of trait empathy such as personal distress. However, insufficient sample size and limited number of *SLC1A1* SNPs may have reduced the likelihood of detecting significant associations. Therefore, further studies with larger sample size and more tag SNPs of the *SLC1A1* gene were warranted.

## Introduction

Although the causes of obsessive compulsive disorder (OCD) are not fully understood, accumulating evidence indicates that the underlying bases of OCD are neurochemical [1]. To date, the serotonin hypothesis is the most prevailing theory regarding the underlying mechanisms of OCD [2]. However, approximately 40% of patients with OCD exhibit an inadequate response to serotonin reuptake inhibitors (SRIs), and functional abnormality of the serotonin system is not always observed in patients with OCD [1]. In addition, research has demonstrated that the serotonin system is closely interrelated with other neurotransmitter systems in the brain [1], indicating that other neurotransmitters may play a role in the pathophysiology of OCD. Recently, several lines of evidence have suggested that alterations in glutamate neurotransmission also contribute to OCD pathophysiology. Magnetic resonance spectroscopy (MRS) studies have implicated dysregulation of glutamate neurotransmission in cortico-striatal-thalamo-cortical circuits in OCD [3]. Additional studies have reported increased levels of glutamate in the cerebrospinal fluid of patients with OCD [4]. Furthermore, the potential therapeutic benefits of some glutamate modulating agents such as memantine [5], N-acetyl cysteine, riluzole [6], and ketamine [7] have been demonstrated in patients with OCD.

Based on the aforementioned evidence, researchers have suggested that genes associated with glutamate neurotransmission may represent the genetic basis of OCD. Among them, *SLC1A1*—which encodes glutamate transporter EAAC1—has received particular attention because several linkage studies have suggested that the 9p24 region, at which *SLC1A1* lies, may contain a susceptibility gene for OCD [8,9]. To date, several studies have investigated the association between *SLC1A1* single nucleotide polymorphisms (SNP) and OCD. Four studies reported a positive association between *SLC1A1* and OCD in single SNP analyses [10–13], while three additional studies reported a significant association between *SLC1A1* and OCD in haplotype analyses, but not in single SNP analyses [14–16]. However, one study found no significant association between two SNPs of *SLC1A1* and OCD, although a significant influence of SNPs on fluoxetine response was observed [17]. A meta-analysis comprising 815 trios, 306 patients with OCD, and 634 controls found no significant influences of *SLC1A1* (nine SNPs) on OCD [18]. These inconsistent findings suggest that additional independent studies are needed. Moreover, to our knowledge, no study has been conducted using subjects of Korean descent regarding the relationship between *SLC1A1* variants and primary OCD, except for studies related to antipsychotics-induced obsessive-compulsive symptoms in schizophrenia [19,20].

Lack of empathy has been regarded as a characteristic trait of OCD [21,22]. Empathy refers to the ability to understand and share others’ emotional states in reference to oneself [23]. Pino et al. [24] suggested that OCD is associated with deficits in understanding the mental and emotional states of others and difficulty sharing in the negative emotional experiences of others. Other studies have also reported that patients with OCD exhibit higher levels of personal distress [21,22], empathic concern [21], and lower perspective taking [22] than controls when trait empathy is measured using the interpersonal reactivity index (IRI). Empathic traits are highly heritable [25], and several studies have suggested that various genes such as oxytocin receptor gene (*OXTR*) [26,27], serotonin receptor gene (*HTR2A*) [28], or brain-derived neurotrophic factor gene (*BDNF*) [29] influence IRI. Because several MRS studies have suggested that the glutamatergic system is associated with empathy [30,31], genes associated with glutamate neurotransmission such as *SLC1A1* represent potential candidates for genetic markers of trait empathy.

Therefore, the present study aimed (1) to replicate the association between *SLC1A1* and OCD in a sample of Korean OCD probands and controls, and (2) to determine the influence of *SLC1A1* on trait empathy as measured using the IRI.

## Materials and methods

### Participants

A total of 615 (man 398, woman 217; mean age 29.9±10.7) unrelated patients with OCD were recruited from Yonsei University Severance Hospital. All patients with OCD were diagnosed using the Structured Clinical Interview and met Axis I criteria specified in the Diagnostic and Statistical Manual of Mental Disorders, Fourth Edition (DSM-IV) [32]. Patients with a lifetime history of any psychotic symptoms, mental retardation, comorbid alcohol or other substance use disorders within the last 6 months, or a history of severe organic or neurologic disorders were excluded. A total of 508 healthy, unrelated individuals (man 286, woman 222; mean age 22.2±2.7) were recruited via community advertisements. All participants were of Korean descent according to their home language and self-identified as being of Korean descent. The institutional review board of Severance Hospital approved the study protocol, and written informed consent was obtained from all participants.

### Measurements

The severity of symptoms in patients with OCD were assessed using the Yale-Brown Obsessive-Compulsive Scale (Y-BOCS) [33]. The severity of depressive symptoms was assessed using the Montgomery-Asberg Depression Rating Scale (MADRS) [34]. Trait empathy was measured using the IRI [35], which contains 28-items scored on a 5-point Likert scale ranging from “Does not describe me well” to “Describes me very well”. The IRI contains four subscales: (1) Perspective Taking (PT), which measures the tendency to adopt the psychological viewpoints of others; (2) Fantasy (FS), which measures the tendency to imagine and identify with fictional characters such as those from books or movies; (3) Empathic Concern (EC), which measures the tendency to experience "other-oriented" feelings of sympathy and concern for the misfortune of others; (4) Personal Distress (PD), which measures the tendency to have "self-oriented" feelings of personal anxiety, discomfort, and unease in tense interpersonal settings [35]. The PD and EC scales assess affective components, whereas the PT scale assesses cognitive components. We used the Korean version of the IRI in the present study [36].

### SNP selection and genotyping

Among all variants of *SLC1A1*, we selected six SNPs (rs2228622, rs3780412, rs301430, rs301434, rs3087879, rs301443) that have been most frequently examined in previous studies and often associated with OCD and/or obsessive-compulsive symptoms in single-marker or haplotype analyses [10–17,19,20,37–40]. Genomic DNA was extracted from blood or saliva. Genotyping was performed using single-base primer extension assay (ABI PRISM SNaPShot Multiplex kit, ABI, Foster City, CA).

### Statistical analyses

Differences in demographic and clinical data between patients and controls were examined using Chi-square (χ^2^) association tests for categorical variables, while *t*-tests or multivariate analyses of variance (ANOVA) were used for continuous variables. Differences in the allelic distribution of the six *SLC1A1* SNPs were examined using χ^2^ tests. Associations between each SNP genotype and OCD status were examined using age- and sex-adjusted multivariate logistic regression analyses. The genetic model was assumed as additive (i.e., genotypes were coded as 0, 1, and 2 based on the minor allele count). Single-marker analyses were performed using the R package SNPassoc [41]. In haplotype analyses, haplotype blocks of the six *SLC1A1* SNPs were defined based on the ‘solid spine of linkage disequilibrium (LD)’ approach in the control sample using Haploview v4.0 [42]. Associations between haplotype distributions and OCD under additive model were examined using the ‘*haplo*.*score*’ function of R package haplo.stats [43], while adjusting for age and sex.

In order to analyze the relationship between *SLC1A1* variants and certain aspects of empathy measured with IRI, we used all available IRI data that were collected from both OCD and control groups. Therefore, we included disease status (affected vs. unaffected) as a covariate, as well as age and sex, in the analyses of IRI data. The influences of each SNP genotype on the four subscale scores of IRI were examined by multivariate logistic regression analyses adjusted for age, sex, and OCD status. The influence of haplotype on each IRI subscale score was examined using the ‘*haplo*.*score*’ function, with adjustment for age, sex, and OCD status.

For single-marker analyses, correction for multiple comparisons of the six SNPs was performed by Bonferroni method. Therefore, the level of statistical significance was set at *p*<0.0083 (0.05/6) for single SNP analysis. For haplotype analyses, permutation adjustments were performed (n = 100,000), and simulated *p*<0.05 was regarded as significant.

The statistical power necessary to identify an association with OCD in the present study was calculated as 84.1–99.7% using Quanto software (version 1.2.4; http://biostats.usc.edu/software), based on the minor allele frequency, a genotype relative risk of 1.5 under the assumption of a log-additive model, and a disease prevalence of 2% [44,45].

## Results

### Demographic and clinical characteristics

The demographic and clinical characteristics of patients with OCD and controls are listed in Table 1. Patients with OCD were significantly older (t = 17.29, *p*<0.001) and more frequently male (χ^2^ = 8.28, *p* = 0.002) than controls. Therefore, we used age and sex as covariates in subsequent analyses. The IRI was assessed from 277 patients with OCD and 395 controls. Patients with OCD exhibited significantly lower PT (F = 5.37, p = 0.02) and higher PD (F = 103.18, *p*<0.001) scores on the IRI than controls.

**Table 1:** Socio-demographic and clinical characteristics of the study sample.

Variable	OCD (n = 615)	Controls (n = 508)	*p*-value
Age, years	29.93 ± 10.71	22.19 ± 2.67	< 0.001
Male/Female, n	398/217	286/222	0.004
Illness duration, years	11.37 ± 8.33		
Y-BOCS score			
Total	24.99 ± 6.00		
Obsessions	12.68 ± 3.04		
Compulsions	12.30 ± 3.47		
Basal MADRS score	18.57 ± 8.69		
Comorbid diagnosis, n (%)			
Major depression	77 (12.5)		
Others	85 (13.8)		
Symptom dimensions, present, n (%)			
Symmetry	476 (77.4)		
Forbidden thoughts	549 (89.3)		
Cleaning	471 (76.6)		
Hoarding	215 (35.0)		
Interpersonal Reactivity Index^a^	(n = 277)	(n = 395)	
Perspective taking	15.13 ± 4.81	15.63 ± 3.58.	0.021
Fantasy seeking	16.26 ± 4.61	16.80 ± 3.88	0.909
Empathic concern	16.62 ± 4.79	15.84 ± 3.58	0.412
Personal distress	16.64 ± 4.97	13.77 ± 3.22	<0.001

OCD, obsessive-compulsive disorder; Y-BOCS, Yale-Brown obsessive-compulsive scale; MADRS, Montgomery-Åsberg depression rating scale; MDD, major depressive disorder.

^a^Multivariate analysis of variance with adjustment of age and sex (Hotelling’s trace; F = 28.36, df = 4, *p*<0.001).

### Association between *SLC1A1* SNPs and OCD status

No SNP data deviated from Hardy–Weinberg equilibrium in the control group (S1 Table). There were no significant associations between allele/genotype of the six SNPs and OCD status (Table 2). In haplotype analyses, two haplotype blocks were identified: block 1 (rs2228622, rs3780412) and block 2 (rs301430, rs301434, rs3087879) (S1 Fig). There were no significant differences in the distribution of block 1 or block 2 haplotype frequencies between the OCD and control groups (Table 3). Considering that significantly different sex ratios between the two groups might have influenced results, we repeated these analyses in male and in female samples separately. However, the results remained unchanged (S2–S5 Tables).

**Table 2:** Distribution of allelic and genotypic frequencies of *SLC1A1* SNPs between OCD and controls.

	Allele				Genotype			
rs number	D/d^a^	OCD^b^	Control^b^	*p-*value^c^	OCD^d^	Control^d^	OR add (95% CI)	*p-*value^e^
rs2228622	G/A	910/314	781/233	0.1426	337/236/39	300/181/26	1.08 (0.86–1.35)	0.5130
rs3780412	T/C	901/327	768/242	0.1491	331/239/44	293/182/30	1.08(0.87–1.35)	0.4795
rs301430	C/T	790/434	660/348	0.6454	253/284/75	218/224/62	0.94(0.77–1.14)	0.5208
rs301434	T/C	1104/122	922/92	0.4816	495/114/4	418/86/3	1.08(0.77–1.50)	0.6638
rs3087879	G/C	1080/144	911/103	0.2272	473/134/5	409/93/5	1.02(0.75–1.40)	0.8980
rs301443	C/G	660/568	572/440	0.1888	182/296/136	176/220/110	1.03(0.85–1.24)	0.7869

OCD, obsessive-compulsive disorder; SNP, single nucleotide polymorphism; OR, odds ratio; CI, confidence interval; add, additive.

^a^Lowercase d denotes the less frequent allele.

^b^Number of major and minor alleles in individuals with OCD and controls.

^c^*p-*values by Pearson’s χ^2^ test for allelic associations.

^d^Number of genotypes in individuals with OCD and controls. Order of genotypes: DD/Dd/dd (d is the minor allele).

^e^*p-*values by multivariate logistic regression, with adjustment for age and sex.

**Table 3:** The effects of *SLC1A1* haplotype on affected status of OCD.

Block			Hap-Freq^a^	Hap-score^b^	Crude *p*^c^	Sim. *p*^d^
1 (rs2228622- rs3780412)^e^						
G	T		0.7276	-0.9275	0.3537	0.3584
A	C		0.2276	0.3112	0.7557	0.7584
G	C		0.0244	1.1025	0.2702	0.2779
A	T		0.0151	1.5637	0.1179	0.1201
2 (rs301430-rs301434-rs3087879)^f^						
T	T	G	0.1578	-1.1051	0.2691	0.2725
T	C	G	0.0846	0.1554	0.8765	0.8774
T	T	C	0.1045	0.1589	0.8737	0.8759
C	C	G	0.0102	0.5159	0.6059	0.6138
C	T	G	0.6285	0.6505	0.5153	0.5195

^a^Hap-Freq, estimated frequency of the haplotype in the pool of all participants.

^b^Hap-Score, score for the haplotype.

^c^Asymptotic chi-square *p*-value.

^d^Simulated *p*-value.

^e^Global-stat = 4.5776, df = 4, *p* = 0.3335, global simulated *p* = 0.3473.

^f^Global-stat = 1.5781, df = 5, *p* = 0.9039, global simulated *p* = 0.9077.

### Influences of *SLC1A1* SNPs on IRI

In single SNP analyses, nominally significant associations were observed between rs301443 and PT (*p* = 0.046), rs3780412 and FS (*p* = 0.044), rs2228622 and PD (*p* = 0.034), and rs3780412 and PD (*p* = 0.016) (S6–S9 Tables). However, the significance of these associations disappeared after adjusting for multiple comparisons. In haplotype analyses, the C-T-G haplotype in block 2 (rs301430-rs301434-rs3087879) was significantly associated with higher PD scores (Hap-score = 2.0427, crude *p* = 0.0411, simulated *p* = 0.0419) (Table 4). However, none of the remaining haplotypes in blocks 1 and 2 exhibited any associations with PT, FS, or EC scores on the IRI (S10–S12 Tables). When the single-marker and haplotype analyses were repeated separately for male and female participants, the association between C-T-G haplotype in block 2 and PD score was no longer significant (data not shown).

**Table 4:** The effects of *SLC1A1* haplotype on personal distress score of IRI.

Block			Hap-Freq^a^	Hap-Score^b^	Crude *p*^c^	Sim. *p*^d^
1 (rs2228622- rs3780412)^e^						
G	T		0.7259	-2.1945	0.0282	0.0283
A	T		0.0137	-0.8380	0.4020	0.4001
G	C		0.0234	0.2688	0.7881	0.7869
A	C		0.2326	2.3562	0.0185	0.0189
2 (rs301430-rs301434-rs3087879)^f^						
T	C	G	0.0810	-1.5968	0.1103	0.1105
T	T	G	0.1538	-1.1870	0.2352	0.2339
T	T	C	0.0986	0.7831	0.4336	0.4344
C	C	G	0.0135	1.0641	0.2873	0.2873
C	T	G	0.6386	2.0427	0.0411	0.0419

IRI, interpersonal reactivity index.

^a^Hap-Freq, estimated frequency of the haplotype in the pool of all subjects.

^b^Hap-Score, score for the haplotype.

^c^Asymptotic chi-square *p*-value.

^d^Simulated *p*-value.

^e^Global-stat = 6.6530, df = 4, *p* = 0.1554, global simulated *p* = 0.1569.

^f^Global-stat = 16.2884, df = 5, *p* = 0.0061, global simulated *p* = 0.0063.

## Discussion

In the present study, we identified no significant associations between six SNPs or haplotypes of *SLC1A1* and OCD in a sample of Korean probands and controls. *SLC1A1* has received attention as a promising candidate gene for OCD, as two independent studies have suggested a linkage between OCD and 9p24, on which *SLC1A1* is located [8,9]. Several subsequent association studies have demonstrated a significant association between *SLC1A1* and OCD. A family-based association study involving 87 patients with early-onset OCD and their parents reported significant associations of rs3780412, rs301430, and the T-C haplotype at rs301430-rs301979 with OCD (in the whole sample and in men but not in women) [11]. Arnold et al. [10] performed a family-based association study involving 157 patients with OCD and 319 of their first-degree relatives, reporting significant associations between OCD and rs301434, rs301435, and rs3087879 SNPs of *SLC1A1*. The authors of this previous study further reported that the C-G haplotype of rs301434-rs3087879 was positively associated with OCD in male offspring. A family-based association study by Stewart et al. [14] involving 66 patients with OCD and their families further reported that the A-T-T haplotype of rs12682807-rs2072657-rs301430 was significantly over-transmitted to offspring with OCD, whereas no single SNPs were associated with OCD. In addition, Shugart et al. [12] performed a family-based association study of 13 *SLC1A1* SNPs in a large sample consisting of 1,950 participants across 378 families (1,006 patients with definite or probable OCD), demonstrating an association between rs301433 and OCD under the dominant model. Wu et al. [16] conducted a case-control study involving 578 patients with OCD and 649 controls among the Han Chinese population. In their study, the G-A-C-G and G-G-T-C haplotypes of rs10491734-rs2228622-rs301430-rs301443 were significantly associated with OCD, although no differences in the distributions of genotypes or alleles for individual SNPs were observed between the OCD and control groups.

Despite such evidence, other studies have reported no influence of *SLC1A1* on OCD. Samuels et al. [13] conducted a family-based association study of 111 SNPs in or near *SLC1A1*. Although several SNPs and a haplotype were nominally significantly associated with OCD, those associations were not significant after correction for multiple comparisons. A recent meta-analysis with 815 trios, 306 patients with OCD, and 634 controls also reported no significant association between *SLC1A1* (nine SNPs) and OCD [18]. Furthermore, Zhang et al. [17] found no association between *SLC1A1* (rs3780412, rs301430) and OCD (OCD, n = 340 and control, n = 350). A case-control study involving patients of the Han Chinese population also reported no association between rs301430 or rs3780412 of *SLC1A1* and OCD [17]. Our findings are in accordance with these studies, suggesting a lack of association between *SLC1A1* SNPs and OCD. This discrepancy between the positive findings of previous studies and our results may be due to differences in the genotype distributions of *SLC1A1* between individuals of European descent and those of Korean descent. For example, Stewart et al. [14] reported a minor allele frequency of 0.43 for rs2228622, whereas this value was 0.23 in the present study (controls). In addition, most studies reporting a positive association between *SLC1A1* and OCD were family-based design, whereas the present study utilized a case-control design. Another possible explanation for this discrepancy is that the haplotypes and LD structure of our study were not identical to those used in previous studies due to differences in the ethnic background of the sample and genotyped SNPs of *SLC1A1*. Indeed, the particular SNPs of *SLC1A1* reported to be associated with OCD are not entirely consistent among studies [10,11,13,14]. Furthermore, in contrast to our study, some previous studies reporting positive associations utilized less conservative methods of correction (e.g., permutations) [10] or failed to perform correction for multiple comparisons [11], potentially leading to false-positive associations. In addition, such inconsistencies regarding the association between *SLC1A1* and OCD may be due to the underlying genetic heterogeneity of OCD.

In the present study, patients with OCD had significantly lower PT and higher PD scores than controls, even after adjusting for age and sex. These findings suggested that patients with OCD exhibit impairments in the ability to spontaneously consider another’s point of view, as well as emotions in response to observing extreme distress in others [35]. Although we mainly aimed to compare IRI scores between the OCD and control groups, our findings of lower PT and higher PD scores in the OCD group are mostly consistent with those of previous studies [21,22]. Interestingly, we observed that the C-T-G haplotype at rs301430-rs301434-rs3087879 of *SLC1A1* was associated with higher PD scores. To our knowledge, the present study is the first to report a genetic influence of *SLC1A1* on trait empathy as measured using the IRI. Although no previous studies have examined the association between *SLC1A1* and empathy, several lines of evidence support the involvement of the glutamatergic system in empathy. One fMRI-MRS study suggested that glutamatergic modulation of emotional processing is critical for inducing feelings of empathy [30]. Another MRS study reported that glutamate concentration in the dorsolateral prefrontal cortex is negatively correlated with PT score on the IRI [31]. Two genetic studies have also demonstrated an influence of the *SLC1A1* gene on autism, which is also characterized by a lack of empathy. A family-based association study involving 86 trios with autism suggested that the T-G haplotype at rs301430-rs301979 is negatively associated with autism (recessive model) [46], while a subsequent study reported that the C allele of rs301430 was associated with more severe anxiety in patients with autism spectrum disorder [47]. One neuroimaging study demonstrated that PD was positively correlated with grey matter volume of the anterior insula and negatively correlated with that in the somatosensory cortex [48]. Research has indicated that glutamate levels in the anterior insula play a major role in emotional processing, and that impairments in glutamate transmission within this region are associated with alexithymia [49]. These findings suggest that *SLC1A1* is associated with trait empathy. However, considering that PD represents affective empathy rather than cognitive empathy, our finding regarding the association between certain haplotypes of *SLC1A1* and PD may be counterintuitive. Therefore, our results should be regarded as preliminary, as further large-scale studies are required to draw more definitive conclusions.

Several limitations of the present study should be addressed. First, controls were not matched with OCD patients in terms of their age and sex. Although the analyses were adjusted for these demographic factors and reanalysis stratified by sex did not reveal significant sex-specific associations, the possibility of confounding effect could not be completely excluded. Second, while our sample size was relatively larger than those of previous studies that examined associations between *SLC1A1* SNPs and OCD, that statistical power of our study findings may not be sufficient for detecting significant associations. Particularly, as IRI was only assessed in a subset of participants, statistical power to find significant relationships between *SLC1A1* variants and IRI scores could have been further reduced and results may have been biased. Hence, results regarding the associations between *SLC1A1* and IRI should be interpreted with caution. Third, all tag SNPs fully covering the *SLC1A1* gene were not included in this study. Although the variants genotyped in this study have been mostly replicated for their associations with OCD and/or obsessive-compulsive symptoms in literature, other variants of *SLC1A1* may be associated with OCD and/or trait empathy in a Korean population. Therefore, we could not draw a clear conclusion regarding the relationship between *SLC1A1* and OCD in a Korean population. Fourth, we did not rule out the possible confounding effects of population substructure, although Korean population is known to be relatively genetically homogeneous. Nonetheless, although we observed no associations between SNPs of *SLC1A1* and OCD in individuals of Korean descent, our findings indicate that a certain haplotype of *SLC1A1* was associated with PD scores, as measured using the IRI. Further studies involving larger sample sizes should investigate a larger number SNPs for the *SLC1A1* gene.

## Supporting information

S1 Fig Linkage disequilibrium (LD) patterns and haplotype blocks estimated with markers that were examined in this study (healthy controls). All numbers in the square represent the pairwise D′ value as a percentile.
(DOCX)

S1 Table Characteristics of SNP markers on the *SLC1A1* gene in controls. (DOCX)

S2 Table Distribution of allelic and genotypic frequencies of *SLC1A1* SNPs between male participants of OCD and control groups. (DOCX)

S3 Table Distribution of allelic and genotypic frequencies of *SLC1A1* SNPs between female participants of OCD and control groups. (DOCX)

S4 Table The effects of *SLC1A1* haplotype on affected status of OCD in male participants. (DOCX)

S5 Table The effects of *SLC1A1* haplotype on affected status of OCD in female participants. (DOCX)

S6 Table The effects of *SLC1A1* SNP on perspective taking score of IRI. (DOCX)

S7 Table The effects of *SLC1A1* SNP on fantasy seeking score of IRI. (DOCX)

S8 Table The effects of *SLC1A1* SNP on empathic concern score of IRI. (DOCX)

S9 Table The effects of *SLC1A1* SNP on personal distress score of IRI. (DOCX)

S10 Table The effects of *SLC1A1* haplotype on perspective taking score of IRI. (DOCX)

S11 Table The effects of *SLC1A1* haplotype on fantasy seeking score of IRI. (DOCX)

S12 Table The effects of *SLC1A1* haplotype on empathic concern score of IRI. (DOCX)

S1 File Dataset. (XLSX)
