# Supplementary material for: Association between glutamate transporter gene polymorphisms and obsessive-compulsive disorder/trait empathy in a Korean population
Source: PLoS One. 2018 Jan 5;13(1):e0190593. doi: 10.1371/journal.pone.0190593 (PMC5755803; doi:10.1371/journal.pone.0190593)

**Figure S1. Linkage disequilibrium (LD) patterns and haplotype blocks estimated with markers that were examined in this study (healthy controls). All numbers in the square represent the pairwise D′ value as a percentile**.


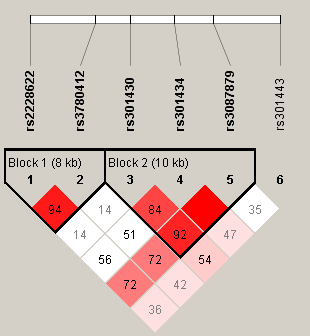

Supplement: S1 Fig — All numbers in the square represent the pairwise D′ value as a percentile. (DOCX) [file pone.0190593.s001.docx]
