# Supplementary material for: Association between glutamate transporter gene polymorphisms and obsessive-compulsive disorder/trait empathy in a Korean population
Source: PLoS One. 2018 Jan 5;13(1):e0190593. doi: 10.1371/journal.pone.0190593 (PMC5755803; doi:10.1371/journal.pone.0190593)
Supplement: S1 Table — (DOCX) [file pone.0190593.s002.docx]

**Table S1. Characteristics of SNP markers on the *SLC1A1* gene in controls.**

| SNP No. | rs No. | Location^a^ | ObsHET | PredHET | p value^b^ | Allele | MAF | Function | Geno^c^ |
| --- | --- | --- | --- | --- | --- | --- | --- | --- | --- |
| 1 | rs2228622 | 4564432 | 0.357 | 0.354 | 0.974 | G>A | 0.230 | Intron, syn. codon | 100.0 |
| 2 | rs3780412 | 4572480 | 0.360 | 0.364 | 0.875 | T>C | 0.240 | Intron | 99.6 |
| 3 | rs301430 | 4576680 | 0.444 | 0.452 | 0.759 | C>T | 0.345 | Intron, syn. codon | 99.4 |
| 4 | rs301434 | 4582082 | 0.170 | 0.165 | 0.776 | T>C | 0.091 | Intron | 100.0 |
| 5 | rs3087879 | 4586808 | 0.183 | 0.183 | 1.000 | G>C | 0.102 | Intron, 3’ UTR | 100.0 |
| 6 | rs301443 | 4594919 | 0.435 | 0.491 | 0.012 | C>G | 0.435 | Intron, nc transcript variant | 99.8 |

HWE, Hardy-Weinberg equilibrium; MAF, minor allele frequency; SNP, single nucleotide polymorphism. rs No. indicates SNP identification in the dbSNP site of NCBI. ObsHET, observed heterozygosity; PredHET, predicted heterozytosity, HWE, Hardy-Weinberg equilibrium; MAF, minor allele frequency; syn.; synonymous; UTR, untranslated region; nc, non-coding

^a^ Information on the chromosomal position is based on NCBI genome build GRCh37.p13.

^b^ *P* value for Hardy-Weinberg equilibrium among controls;

^c^ Genotyping call rate (%).
