# Supplementary material for: Association between glutamate transporter gene polymorphisms and obsessive-compulsive disorder/trait empathy in a Korean population
Source: PLoS One. 2018 Jan 5;13(1):e0190593. doi: 10.1371/journal.pone.0190593 (PMC5755803; doi:10.1371/journal.pone.0190593)
Supplement: S2 Table — (DOCX) [file pone.0190593.s003.docx]

**S2 Table. Distribution of allelic and genotypic frequencies of *SLC1A1* SNPs between male participants of OCD and control group.**

|  | Allele | | | | Genotype | | | |
| --- | --- | --- | --- | --- | --- | --- | --- | --- |
| rs number | D/d^a^ | OCD^b^ | Control^b^ | *p*^c^ | OCD^d^ | Control^d^ | OR add  (95% CI) | *p*^e^ |
| rs2228622 | G/A | 587/203 | 439/131 | 0.2513 | 221/145/29 | 169/101/15 | 1.08 (0.82-1.41) | 0.5815 |
| rs3780412 | T/C | 584/210 | 427/139 | 0.4315 | 220/144/33 | 162/103/18 | 1.03(0.79-1.34) | 0.8171 |
| rs301430 | C/T | 510/284 | 358/210 | 0.6487 | 163/184/50 | 117/124/43 | 0.90(0.71-1.14) | 0.3654 |
| rs301434 | T/C | 702/90 | 517/53 | 0.2200 | 309/84/3 | 233/51/1 | 1.27(0.86-1.88) | 0.2361 |
| rs3087879 | G/C | 707/87 | 513/57 | 0.5704 | 313/81/3 | 232/49/4 | 0.94(0.64-1.38) | 0.7556 |
| rs301443 | C/G | 426/368 | 322/246 | 0.2666 | 120/186/91 | 100/122/62 | 1.08(0.86-1.34) | 0.5257 |

OCD, obsessive-compulsive disorder; SNP, single nucleotide polymorphism; OR, odds ratio; CI, confidence interval; add, additive.

^a^Lowercase d denotes the less frequent allele. ^b^Minor allele frequencies in individuals with OCD and controls. ^c^*p-*values by Pearson’s χ^2^ test for allelic associations. ^d^Number of genotypes in individuals with OCD and controls. Order of genotypes: DD/Dd/dd (d is the minor allele). ^e^*p-*values by multivariate logistic regression, with adjustment for age and sex.
