# Supplementary material for: Association between glutamate transporter gene polymorphisms and obsessive-compulsive disorder/trait empathy in a Korean population
Source: PLoS One. 2018 Jan 5;13(1):e0190593. doi: 10.1371/journal.pone.0190593 (PMC5755803; doi:10.1371/journal.pone.0190593)
Supplement: S3 Table — (DOCX) [file pone.0190593.s004.docx]

**S3 Table. Distribution of allelic and genotypic frequencies of *SLC1A1* SNPs between female participants of OCD and control group.**

|  | Allele | | | | Genotype | | | |
| --- | --- | --- | --- | --- | --- | --- | --- | --- |
| rs number | D/d^a^ | OCD^b^ | Control^b^ | *p*^c^ | OCD^d^ | Control^d^ | OR add  (95% CI) | *p*^e^ |
| rs2228622 | G/A | 323/111 | 342/102 | 0.3683 | 116/91/10 | 131/80/11 | 1.10 (0.73-1.67) | 0.6456 |
| rs3780412 | T/C | 317/117 | 341/103 | 0.1986 | 111/95/11 | 131/79/12 | 1.22(0.81-1.83) | 0.3483 |
| rs301430 | C/T | 280/150 | 302/138 | 0.2700 | 90/100/25 | 101/100/19 | 1.02(0.69-1.50) | 0.9271 |
| rs301434 | T/C | 402/32 | 405/39 | 0.4434 | 186/30/1 | 185/35/2 | 0.68(0.35-1.32) | 0.2439 |
| rs3087879 | G/C | 373/57 | 398/46 | 0.1844 | 160/53/2 | 177/44/1 | 1.24(0.71-2.17) | 0.4489 |
| rs301443 | C/G | 234/200 | 250/194 | 0.4767 | 62/110/45 | 76/98/48 | 0.90(0.64-1.28) | 0.5747 |

OCD, obsessive-compulsive disorder; SNP, single nucleotide polymorphism; OR, odds ratio; CI, confidence interval; add, additive.

^a^Lowercase d denotes the less frequent allele. ^b^Minor allele frequencies in individuals with OCD and controls. ^c^*p-*values by Pearson’s χ^2^ test for allelic associations. ^d^Number of genotypes in individuals with OCD and controls. Order of genotypes: DD/Dd/dd (d is the minor allele). ^e^*p-*values by multivariate logistic regression, with adjustment for age and sex.
