# Supplementary material for: Association between glutamate transporter gene polymorphisms and obsessive-compulsive disorder/trait empathy in a Korean population
Source: PLoS One. 2018 Jan 5;13(1):e0190593. doi: 10.1371/journal.pone.0190593 (PMC5755803; doi:10.1371/journal.pone.0190593)
Supplement: S4 Table — (DOCX) [file pone.0190593.s005.docx]

**S4 Table. The effects of *SLC1A1* haplotype on affected status of OCD in male participants.**

| ` | | | Hap-Freq^a^ | Hap-score^b^ | Crude *p*^c^ | Sim. *p*^d^ |
| --- | --- | --- | --- | --- | --- | --- |
| 1 (rs2228622- rs3780412)^e^ | | |  |  |  |  |
| G | T |  | 0.7233 | -0.4355 | 0.6632 | 0.6664 |
| G | C |  | 0.0252 | -0.0197 | 0.9843 | 0.9845 |
| A | C |  | 0.2277 | 0.2220 | 0.8243 | 0.8246 |
| A | T |  | 0.0150 | 1.5314 | 0.1257 | 0.1303 |
| 2 (rs301430-rs301434-rs3087879)^f^ | | |  |  |  |  |
| T | T | G | 0.1674 | -1.4489 | 0.1474 | 0.1531 |
| T | T | C | 0.1004 | -0.2631 | 0.7924 | 0.7939 |
| T | C | G | 0.0919 | 0.8184 | 0.4131 | 0.4183 |
| C | T | G | 0.6155 | 0.8192 | 0.4127 | 0.4170 |
| C | C | G | 0.0126 | 1.2762 | 0.2019 | 0.2111 |

^a^Hap-Freq, estimated frequency of the haplotype in the pool of all male participants. ^b^Hap-Score, score for the haplotype. ^c^Asymptotic chi-square *p*-value. ^d^Simulated *p*-value. ^e^Global-stat=2.8379, df=4, *p*=0.5853, global simulated *p*=0.6046. ^f^Global-stat=5.3763, df=5, *p*=0.3717, global simulated *p*=0.3881.
