# Supplementary material for: Association between glutamate transporter gene polymorphisms and obsessive-compulsive disorder/trait empathy in a Korean population
Source: PLoS One. 2018 Jan 5;13(1):e0190593. doi: 10.1371/journal.pone.0190593 (PMC5755803; doi:10.1371/journal.pone.0190593)
Supplement: S5 Table — (DOCX) [file pone.0190593.s006.docx]

**S5 Table. The effects of *SLC1A1* haplotype on affected status of OCD in female participants.**

| ` | | | Hap-Freq^a^ | Hap-score^b^ | Crude *p*^c^ | Sim. *p*^d^ |
| --- | --- | --- | --- | --- | --- | --- |
| 1 (rs2228622- rs3780412)^e^ | | |  |  |  |  |
| G | T |  | 0.7343 | -1.0392 | 0.2987 | 0.2935 |
| G | C |  | 0.2274 | 0.3416 | 0.7327 | 0.7292 |
| A | T |  | 0.0152 | 0.4711 | 0.6376 | 0.6400 |
| G | C |  | 0.0232 | 1.6724 | 0.0944 | 0.0899 |
| 2 (rs301430-rs301434-rs3087879)^f^ | | |  |  |  |  |
| T | C | G | 0.0731 | -1.1651 | 0.2440 | 0.2379 |
| C | C | G | 0.0066 | -1.1369 | 0.2556 | 0.2486 |
| C | T | G | 0.6487 | -0.0073 | 0.9942 | 0.9942 |
| T | T | G | 0.1429 | 0.0364 | 0.9709 | 0.9711 |
|  | T | G | 0.0068 | 0.0507 | 0.9596 | 0.9714 |
| T | T | C | 0.1109 | 0.7659 | 0.4438 | 0.4404 |

^a^Hap-Freq, estimated frequency of the haplotype in the pool of all female participants. ^b^Hap-Score, score for the haplotype. ^c^Asymptotic chi-square *p*-value. ^d^Simulated *p*-value. ^e^Global-stat=3.2845, df=3, *p*=0.3498, global simulated *p*=0.3414. ^f^Global-stat=4.4074, df=6, *p*=0.6217, global simulated *p*=0.6060.
