# Supplementary material for: Association between glutamate transporter gene polymorphisms and obsessive-compulsive disorder/trait empathy in a Korean population
Source: PLoS One. 2018 Jan 5;13(1):e0190593. doi: 10.1371/journal.pone.0190593 (PMC5755803; doi:10.1371/journal.pone.0190593)
Supplement: S6 Table — (DOCX) [file pone.0190593.s007.docx]

**Table S6. The effects of *SLC1A1* SNP on perspective taking score of IRI.**

| rs number | D/d^a^ | DD/Dd/dd^b^ | DD^c^ | Dd^c^ | dd^c^ | Mean difference  (95% CI) | *p*^d^ |
| --- | --- | --- | --- | --- | --- | --- | --- |
| rs2228622 | G/A | 376/256/38 | 15.21 ± 0.21 | 15.69 ± 0.26 | 15.53 ±0.61 | 0.3227(-0.20-0.84) | 0.2240 |
| rs3780412 | T/C | 367/261/42 | 15.29 ± 0.21 | 15.64 ± 0.26 | 15.14 ± 0.58 | 0.1471(-0.36-0.66) | 0.5731 |
| rs301430 | C/T | 294/298/76 | 15.47 ± 0.24 | 15.19 ± 0.24 | 15.99 ± 0.45 | 0.0784(-0.39-0.55) | 0.7432 |
| rs301434 | T/C | 547/118/5 | 15.37 ± 0.18 | 15.68 ± 0.36 | 15.00 ± 1.73 | 0.2654(-0.50-1.03) | 0.4941 |
| rs3087879 | G/C | 536/128/5 | 15.38 ± 0.18 | 15.51 ± 0.40 | 17.60 ± 1.47 | 0.2606(-0.48-1.00) | 0.4909 |
| rs301443 | C/G | 223/301/147 | 15.00 ± 0.28 | 15.53 ± 0.24 | 15.84 ± 0.31 | 0.4333(0.01-0.86) | 0.0462 |

IRI, interpersonal reactivity index; SNP, single nucleotide polymorphism; OR, odds ratio; CI, confidence interval; add, additive.

^a^Lowercase d denotes the less frequent allele.

^b^Number of genotypes

^c^ mean ± standard error

^d^*p* values by multivariate logistic regression, with adjustment for age, sex, and affected status
