# Supplementary material for: Association between glutamate transporter gene polymorphisms and obsessive-compulsive disorder/trait empathy in a Korean population
Source: PLoS One. 2018 Jan 5;13(1):e0190593. doi: 10.1371/journal.pone.0190593 (PMC5755803; doi:10.1371/journal.pone.0190593)
Supplement: S7 Table — (DOCX) [file pone.0190593.s008.docx]

**Table S7. The effects of *SLC1A1* SNP on fantasy seeking score of IRI.**

| rs number | D/d^a^ | DD/Dd/dd^b^ | DD^c^ | Dd^c^ | dd^c^ | Mean difference  (95% CI) | *p*^d^ |
| --- | --- | --- | --- | --- | --- | --- | --- |
| rs2228622 | G/A | 376/256/38 | 16.41 ± 0.21 | 16.70 ± 0.28 | 17.50 ±0.56 | 0.4913(-0.04-1.02) | 0.0678 |
| rs3780412 | T/C | 367/261/42 | 16.43 ± 0.22 | 16.52 ± 0.27 | 18.17 ± 0.58 | 0.5305(0.01-1.05) | 0.0443 |
| rs301430 | C/T | 294/298/76 | 16.69 ± 0.25 | 16.56 ± 0.24 | 16.24 ± 0.46 | -0.1895(-0.66-0.28) | 0.4338 |
| rs301434 | T/C | 547/118/5 | 16.59 ± 0.18 | 16.52 ± 0.39 | 19.20 ± 1.93 | 0.1621(-0.61-0.93) | 0.6804 |
| rs3087879 | G/C | 536/128/5 | 16.53 ± 0.18 | 16.78 ± 0.39 | 18.00 ± 1.70 | 0.3690(-0.38-1.12) | 0.3344 |
| rs301443 | C/G | 223/301/147 | 16.75 ± 0.27 | 16.27 ± 0.25 | 16.99 ± 0.34 | 0.1154(-0.32-0.55) | 0.6010 |

IRI, interpersonal reactivity index; SNP, single nucleotide polymorphism; OR, odds ratio; CI, confidence interval; add, additive.

^a^Lowercase d denotes the less frequent allele.

^b^Number of genotypes

^c^ mean ± standard error

^d^*p* values by multivariate logistic regression, with adjustment for age, sex, and affected status
