# Supplementary material for: Association between glutamate transporter gene polymorphisms and obsessive-compulsive disorder/trait empathy in a Korean population
Source: PLoS One. 2018 Jan 5;13(1):e0190593. doi: 10.1371/journal.pone.0190593 (PMC5755803; doi:10.1371/journal.pone.0190593)
Supplement: S8 Table — (DOCX) [file pone.0190593.s009.docx]

**Table S8. The effects of *SLC1A1* SNP on empathic concern score of IRI.**

| rs number | D/d^a^ | DD/Dd/dd^b^ | DD^c^ | Dd^c^ | dd^c^ | Mean difference (95% CI) | *p*^d^ |
| --- | --- | --- | --- | --- | --- | --- | --- |
| rs2228622 | G/A | 376/256/38 | 15.90 ± 0.22 | 16.49 ± 0.24 | 16.42 ±0.61 | 0.3411(-0.18-0.86) | 0.1974 |
| rs3780412 | T/C | 367/261/42 | 15.92 ± 0.22 | 16.36 ± 0.25 | 16.62 ± 0.55 | 0.2896(-0.22-0.80) | 0.2652 |
| rs301430 | C/T | 294/298/76 | 16.21 ± 0.25 | 15.92 ± 0.23 | 16.86 ± 0.47 | 0.1219(-0.35-0.59) | 0.6091 |
| rs301434 | T/C | 547/118/5 | 16.04 ± 0.18 | 16.68 ± 0.34 | 17.60 ± 2.11 | 0.6623(-0.09-1.42) | 0.0863 |
| rs3087879 | G/C | 536/128/5 | 16.11 ± 0.18 | 16.38 ± 0.35 | 17.60 ± 1.08 | 0.2741(-0.46-1.01) | 0.4660 |
| rs301443 | C/G | 223/301/147 | 15.98 ± 0.27 | 15.99 ± 0.25 | 16.79 ± 0.32 | 0.3056(-0.12-0.73) | 0.1584 |

IRI, interpersonal reactivity index; SNP, single nucleotide polymorphism; OR, odds ratio; CI, confidence interval; add, additive.

^a^Lowercase d denotes the less frequent allele.

^b^Number of genotypes

^c^ mean ± standard error

^d^*p* values by multivariate logistic regression, with adjustment for age, sex, and affected status
