# Supplementary material for: Association between glutamate transporter gene polymorphisms and obsessive-compulsive disorder/trait empathy in a Korean population
Source: PLoS One. 2018 Jan 5;13(1):e0190593. doi: 10.1371/journal.pone.0190593 (PMC5755803; doi:10.1371/journal.pone.0190593)
Supplement: S9 Table — (DOCX) [file pone.0190593.s010.docx]

**Table S9. The effects of *SLC1A1* SNP on personal distress score of IRI.**

| rs number | D/d^a^ | DD/Dd/dd^b^ | DD^c^ | Dd^c^ | dd^c^ | Mean difference (95% CI) | *p*^d^ |
| --- | --- | --- | --- | --- | --- | --- | --- |
| rs2228622 | G/A | 376/256/38 | 14.64 ± 0.23 | 15.24 ± 0.25 | 16.08 ± 0.69 | 0.5431(0.04-1.04) | 0.0342 |
| rs3780412 | T/C | 367/261/42 | 14.60 ± 0.23 | 15.28 ± 0.25 | 16.14 ± 0.68 | 0.6058(0.11-1.10) | 0.0161 |
| rs301430 | C/T | 294/298/76 | 15.33 ± 0.25 | 14.73 ± 0.25 | 14.55 ± 0.45 | -0.3958(-0.85-0.06) | 0.0861 |
| rs301434 | T/C | 547/118/5 | 15.09 ± 0.18 | 14.36 ± 0.38 | 17.00 ± 1.92 | -0.5102(-1.24-0.22) | 0.1710 |
| rs3087879 | G/C | 536/128/5 | 14.90 ± 0.19 | 15.26 ± 0.36 | 14.40 ± 1.50 | 0.1840(-0.53-0.90) | 0.6131 |
| rs301443 | C/G | 223/301/147 | 14.91 ± 0.30 | 14.93 ± 0.23 | 15.10 ± 0.38 | -0.006(-0.42-0.41) | 0.9764 |

IRI, interpersonal reactivity index; SNP, single nucleotide polymorphism; OR, odds ratio; CI, confidence interval; add, additive.

^a^Lowercase d denotes the less frequent allele.

^b^Number of genotypes

^c^ mean ± standard error

^d^*p* values by multivariate logistic regression, with adjustment for age, sex, and affected status
