# Supplementary material for: Association between glutamate transporter gene polymorphisms and obsessive-compulsive disorder/trait empathy in a Korean population
Source: PLoS One. 2018 Jan 5;13(1):e0190593. doi: 10.1371/journal.pone.0190593 (PMC5755803; doi:10.1371/journal.pone.0190593)
Supplement: S10 Table — (DOCX) [file pone.0190593.s011.docx]

**Table S10. The effects of *SLC1A1* haplotype on perspective taking score of IRI.**

| Block | | | Hap-Freq^a^ | Hap-Score^b^ | Crude *p*^c^ | Sim. *p*^d^ |
| --- | --- | --- | --- | --- | --- | --- |
| 1 (rs2228622- rs3780412)^e^ | | |  |  |  |  |
| G | C |  | 0.0234 | -1.6096 | 0.1075 | 0.1075 |
| G | T |  | 0.7259 | -0.8533 | 0.3935 | 0.3957 |
| A | T |  | 0.0137 | 0.4042 | 0.6860 | 0.6853 |
| A | C |  | 0.2326 | 1.0365 | 0.3000 | 0.3022 |
| 2 (rs301430-rs301434-rs3087879)^f^ | | |  |  |  |  |
| C | T | G | 0.6386 | -0.8420 | 0.3998 | 0.3976 |
| T | T | G | 0.1538 | -0.4414 | 0.6590 | 0.6581 |
| T | C | G | 0.0810 | 0.1330 | 0.8942 | 0.8926 |
| T | T | C | 0.0986 | 0.7527 | 0.4516 | 0.4554 |
| C | C | G | 0.0135 | 1.6207 | 0.1051 | 0.1050 |

IRI, interpersonal reactivity index

^a^ Hap-Freq, estimated frequency of the haplotype in the pool of all subjects; ^b^ Hap-Score, score for the haplotype; ^c^ asymptotic chi-square *p*-value (haplotype *p*); ^d^ simulated *p*-value; ^e^ global-stat=5.9738, df=4, *p*=0.2011, global simulation *p*=0.1997; ^f^ global-stat=4.2685, df=5, *p*=0.5115, global simulation *p*=0.5134.
