# Supplementary material for: Association between glutamate transporter gene polymorphisms and obsessive-compulsive disorder/trait empathy in a Korean population
Source: PLoS One. 2018 Jan 5;13(1):e0190593. doi: 10.1371/journal.pone.0190593 (PMC5755803; doi:10.1371/journal.pone.0190593)
Supplement: S11 Table — (DOCX) [file pone.0190593.s012.docx]

**Table S11. The effects of *SLC1A1* haplotype on fantasy seeking score of IRI.**

| Block | | | Hap-Freq^a^ | Hap-Score^b^ | Crude *p*^c^ | Sim. *p*^d^ |
| --- | --- | --- | --- | --- | --- | --- |
| 1 (rs2228622- rs3780412)^*^ | | |  |  |  |  |
| G | T |  | 0.7259 | -2.1073 | 0.0351 | 0.0368 |
| A | T |  | 0.0137 | 0. 2421 | 0.8087 | 0.8076 |
| G | C |  | 0.0234 | 0.8848 | 0.3763 | 0.3763 |
| A | C |  | 0.2326 | 1.7035 | 0.0885 | 0.0909 |
| 2 (rs301430-rs301434-rs3087879)^**^ | | |  |  |  |  |
| T | T | G | 0.1538 | -2.0214 | 0.0432 | 0.0423 |
| T | C | G | 0.0810 | -0.1116 | 0.9111 | 0.9119 |
| C | T | G | 0.6386 | 0.8140 | 0.4157 | 0.4172 |
| T | T | C | 0.0986 | 1.2594 | 0.2079 | 0.2068 |
| C | C | G | 0.0135 | 1.2920 | 0.1964 | 0.1940 |

IRI, interpersonal reactivity index

^a^ Hap-Freq, estimated frequency of the haplotype in the pool of all subjects; ^b^ Hap-Score, score for the haplotype; ^c^ asymptotic chi-square *p*-value (haplotype *p*); ^d^ simulated *p*-value; ^e^ global-stat=4.7647, df=4, *p*=0.3123, global simulation *p*=0.3136; ^f^ global-stat=7.4553, df=5, *p*=0.1889, global simulation *p*=0.1883.
