# Supplementary material for: Association between glutamate transporter gene polymorphisms and obsessive-compulsive disorder/trait empathy in a Korean population
Source: PLoS One. 2018 Jan 5;13(1):e0190593. doi: 10.1371/journal.pone.0190593 (PMC5755803; doi:10.1371/journal.pone.0190593)
Supplement: S12 Table — (DOCX) [file pone.0190593.s013.docx]

**Table S12. The effects of *SLC1A1* haplotype on empathic concern score of IRI.**

| Block | | | Hap-Freq^a^ | Hap-Score^b^ | Crude *p*^c^ | Sim. *p*^d^ |
| --- | --- | --- | --- | --- | --- | --- |
| 1 (rs2228622- rs3780412)^*^ | | |  |  |  |  |
| G | C |  | 0.0234 | -1.3783 | 0.1681 | 0.1687 |
| A | T |  | 0.0137 | -1.1475 | 0.2512 | 0.2487 |
| G | T |  | 0.7259 | -0.7944 | 0.4270 | 0.4275 |
| A | C |  | 0.2326 | 1.6332 | 0.1024 | 0.1026 |
| 2 (rs301430-rs301434-rs3087879)^**^ | | |  |  |  |  |
| T | T | G | 0.1538 | -1.0418 | 0.2975 | 0.2982 |
| C | T | G | 0.6386 | -0.5922 | 0.5537 | 0.5544 |
| T | T | C | 0.0986 | 0.9083 | 0.3637 | 0.3624 |
| C | C | G | 0.0135 | 1.3433 | 0.1792 | 0.1783 |
| T | C | G | 0.0810 | 1.5259 | 0.1270 | 0.1276 |

IRI, interpersonal reactivity index

^a^ Hap-Freq, estimated frequency of the haplotype in the pool of all subjects; ^b^ Hap-Score, score for the haplotype; ^c^ asymptotic chi-square *p*-value (haplotype p); ^d^ simulated *p*-value; ^e^ global-stat=5.5446, df=4, *p*=0.2358, global simulation *p*=0.2361; ^f^ global-stat=6.6923, df=5, *p*=0.2446, global simulation *p*=0.2455
